# Extracellular NM23 Signaling in Breast Cancer: *Incommodus Verum*

**DOI:** 10.3390/cancers3032844

**Published:** 2011-07-06

**Authors:** Iain L.O. Buxton, Nucharee Yokdang

**Affiliations:** Department of Pharmacology, University of Nevada School of Medicine, Center for Molecular Medicine, Mail Stop 573, 1664 N. Virginia Street, Reno, NV 89557, USA; E-Mail: nyokdang@medicine.nevada.edu (N.Y.)

**Keywords:** breast cancer, dormancy, latency, NDPK, nucleotides, purinergic receptors, signaling, angiogenesis

## Abstract

The notion that breast cancers can survive in an individual patient in a dormant state only to grow as metastatic disease in the future, is in our view incontrovertibly established. Convincing too is the evidence that surgery to remove the primary tumor often terminates dormancy resulting in accelerated relapses. Accepting that many deaths due to breast cancer might be averted were we to understand the cellular mechanisms underlying escape from dormancy, we have examined the extracellular signals produced by breast cancers derived from women with metastatic breast disease. In this perspective, we explore the role of extracellular nucleotide signaling that we have proposed constitutes a pathological axis from the transformed tumor cell to the endothelium in the service of intravasation, dissemination, extravasation and angiogenesis. A role for the dinucleotide kinase NM23/NDPK (nucleoside diphosphate kinase) secreted by breast tumor cells in the generation of signals that stimulate vascular leakiness, anti-thrombosis, endothelial migration and growth, constitutes a mechanistic basis for escape from latency and offers putative therapeutic targets for breast cancer management not previously appreciated.

## 1. Introduction

The surgical imperative to remove a primary carcinoma of the breast in a newly diagnosed patient dominates the current treatment approach and sets the stage for the emotion that has overtaken the science underlying our understanding of breast cancer progression. This imperative may be flawed. Whether reviewed from the historical perspective so thoroughly offered by Retsky and colleagues [1], or viewed from current survival data, powerful evidence supports the view that dissemination of breast cancer cells that may become metastases has already occurred before clinical or radiological detection in about 90% of all breast cancers as reasoned by Retsky [1]. Surely this is indeed an “inconvenient truth” [2] (*incommodus verum*) since the assumption that “catching” cancer early will offer a better outcome for the patient does not fit with the facts.

While the notion of leaving a primary breast cancer lesion *in situ* may seem heretical, a better understanding of tumor biology might redefine what could be done before, or concurrent with surgical removal that might establish continued suppression of those cells dormant at the time of surgery. The hazard from primary tumor removal to subsequent metastatic disease has been established in patients untreated following surgery [1,3,4]. The fact that primary tumors produce mediators that suppress their own metastases provided a “*cure*” for breast cancer in mouse models of the human disease [5-7]. Such mechanisms may lie at the root of dormancy [8] such that removal of the primary advantages the growth of metastases.

The ability to find ductal carcinoma *in situ* (DCIS) by mammography has exceeded our diagnostic grasp. A large proportion of such cases are false positives (perhaps as large as 20% [9]) and others might never result in disease. This is no small matter since a woman hearing that she may have “breast cancer” will be hit by a cruel emotional rollercoaster if the DCIS turns out to be merely calcium. If not a false alarm, what can we say with certainty will be her fate? While magnetic resonance imaging of the breast and now the 3-dimensional mammogram are of technical marvel [10], their contribution to keeping women alive is debatable [11].

If breast cancer cells have left the site of the original transformed lesion to reside elsewhere in the body, but not produce disease for many years, then it is critically important to determine what events permit their passage to other sites in the body; why once disseminated they do not continue to grow, only to lie dormant; and what triggers their exit from dormancy; and once this change occurs, what biologic processes transpire that support their growth as metastases?

While these events will be the result of disparate pathways guiding the interaction of transformed cells with normal compartments such as capillary beds in tissues such as the lung, we suggest that some of these answers lie in a better understanding of the actions of extracellular NM23/NDPK (nucleoside diphosphate kinase) shed from breast cancer cells. In the following discussion, we will review recent work from our laboratory and that of others that sheds light on these questions and offers a challenge to our current view of tumor biology.

## 2. Discussion

### 2.1. Early Dissemination

While dissemination of breast disease to regional lymphatics has been used both for diagnostic and treatment objectives, it is clear that clinically important breast cancer metastases to which patients succumb, do not establish as a result of the passage of breast cancer cells via lymph. Indeed, perhaps the best proof for this comes from the surgical approaches taken in the past to provide a surgical “cure” for breast cancer [12]. The radical mastectomy which removed the entire breast and its lymph drainage did nothing to reduce the outcomes for women, let alone cure the disease as was supposed at the time [13]. It is clear in retrospect that this approach was based on the simplest of notions formed on a surgical bias that if all disease is removed from the breast, the patient will be cured. So if metastases can occur in women at early stage breast cancer treated by radical means [14-16], then it stands to reason that lymph is not the primary portal for metastatic dissemination and that the cells that will give rise to metastatic disease many years later have already arrived elsewhere at the time of diagnosis. The answer of course is that breast cancer cells gain access to and disseminate via the blood stream [17]. How cancer cells can accomplish this is unclear.

If cells move early and can, but do not always generate metastatic disease years later, then there are but two inescapable possibilities. One is that the phenotype of the cells that constitute the events of dissemination are disparate, if overlapping with those that support metastatic disease [18] and thus, the transforming events that generate metastatic disease do not accumulate readily. The second notion, that may be a subset of the first, is that the cells that disseminate early are prevented from growing as disease by factors secreted by the primary tumor. Transformations of breast cells that confer motility [19], intravasation and extravasation may be properties that are either transiently expressed, turned off when cells reach a distant site, or are insufficient for metastatic growth such that a second transforming event or events must accumulate to trigger frank metastasis.

In our view, an interesting way to approach the question of metastasis is to ask if breast cancer cells once mobile (this too is not obvious), mimic or even enlist the actions of cells that regularly enter the tissue from the blood stream. In simple terms, intravasation and extravasation of breast cancer cells may be the result of their ability to mimic leukocytes that regularly exit the blood stream to fight infection. The effects of VEGFR2 activation that render capillary vessels permeable [20], particularly those that feed the primary tumor [21], or even the effect of purinergic receptor agonists to promote the effects of VEGFR2 could support intravasation. If one imagines that breast cancer cells enter the blood stream in numbers sufficient to populate several tissue beds such as bone, brain and lung [22], then it stands to reason that cells must do more than get in and get out of capillaries. These invaders must also be able to evade immune mechanisms that would remove them, prevent the formation of activated platelets that could prevent their passage and dilate arterioles so that they can move easily into capillary beds.

While a detailed examination of the ability of breast tumors to evade immune recognition is beyond the scope of this perspective, the subject has been examined and thought to relate to the ability of cells to immunoedit [23]. Tumors evade the immune system following immune surveillance by either directly inducing tolerance or by altering their phenotype to suppress or evade immunity. This latter mechanism has been termed immunoediting [24,25] which can be induced pharmacologically [24,26] and results in cellular reprogramming that may be accompanied by alterations in tumor characteristics including increased invasive potential associated with epithelial to mesenchymal transition [27].

Whatever the precise details of the epithelial to mesenchymal transition (EMT), it appears that these genetic changes are sufficient to constitute invasive characteristics [28] that permit tumor cells to be present at capillary sites for intravasation. Intravasation is thought to require macrophage association with endothelium that together with the tumor cell, constitutes a microenvironment suitable for intravasation [29]. We propose that there is one additional factor present, tumor secreted NM23/NDPK.

### 2.2. MN23/NDPK

NM23 is a nucleoside diphosphate kinase (NDPK) regenerating ATP (adenosine 5′-triphosphate) levels for intracellular “housekeeping” enzymes by covalently transferring the γ-phosphate from a nucleoside triphosphate (NTP) such as GTP (guanosine 5′-triphosphate), to a nucleoside diphosphate acceptor (NDP; e.g., ADP (adenosine 5′-diphosphate)) via a ping-pong transphosphorylase mechanism [30]. In this role, NDPK serves to maintain ATP levels. NM23/NDPK has also been shown to act as a histidine kinase, a transcription activator and an exonuclease [31]. NM23/NDPK is clearly a multifunctional protein distributed in the cytosol and plasma membrane, as well as the nucleus [32] where the NDPK-B (NM23-h2) isoform functions as PuF, a c-MYC transcription factor [31]. NM23 was originally described as non-metastatic 23 gene, which was found in mouse carcinoma cells as a homolog of the drosophila *awd* protein (altered wing disk) whose expression was thought to be inversely related to metastasis potential [33,34] although this has been shown to be less straightforward than originally thought [31,35-37].

Contradictory evidence regarding NDPK-induced metastasis inhibition has been found in that RNA (ribonucleic acid) levels of H1, and H2 are elevated in aggressive neuroblastoma and colorectal cancers [38-41]. There is more evidence to support an intracellular role for NM23/NDPK in tumor metastasis [34,42,43] as well as finding high tissue levels of NDPK-A protein in patients with breast carcinoma [44-47]. Hamby *et al.* confirmed that the catalytically inactive H118Y mutant of NDPK-B significantly suppressed the lung metastasis of human melanoma cells *in vivo*. Because mutations in NM23 genes are rare in cancer [31], NM23 may be an example of a family of cancer genes that become dysregulated not through mutation (despite its description as a tumor suppressor gene [48]), but through expression changes at the protein level.

A pathological role of extracellular NDPK-A and B is suggested in multiple studies of patient serum where NDPK-A is overexpressed as in extracellular fluid from non-Hodgkin lymphoma [49,50], neuroblastoma [51], T and B-cell lymphoma [52,53], the bronchial system of patients with squamous cell lung cancer [54] and NDPK-A serum levels from patients with various hematological malignancies when compared to normal serum [55]. The serum level of NM23-H1 protein was clinically useful as a prognostic factor in malignant lymphoma and acute myelogenous leukemia [49,56]. In *in vitro* studies, extracellular NDPK-B was secreted by MDA-MB-435 breast cancer, colon and pancreas cell lines [57], while NDPK-A was secreted by human leukemia cell lines [58] into the extracellular enviroment. The study established that NDPK-A promotes growth of acute myelogenous leukemia cells [59,60], and that NDPKs promote endothelial cells tube formation *in vitro* [61].

For an NDPK to appear in the blood stream suggests that it would likely act to generate ATP from ADP in the blood stream were a phosphoryl donor present. It is known that nucleotides appear normally in the blood stream [62] and that there is a likely mechanistic role for nucleotides in regulating blood flow on a moment-to-moment basis in certain vascular beds [63-65]. The hypothesis that there is a role for a shed or secreted form of NM23/NDPK subserving breast cancer development has suffered from the NM23 dogma of Steeg [66] and others if the staunch criticism we have received is any measure. Nonetheless, it is clear from *in vitro* [61,67,68] and recent *in vivo* work from our lab [69] together with published work from others [34,45-47] that secreted NM23 contributes to our understanding of metastatic potential.

Expression of a cell-surface NDPK activity is known to regulate extracellular nucleotide levels in various cell types [70-72]. A catalytically inactive NDPK mutant (H118Y) made by Hamby and colleagues confirmed that NDPK acting as an NDP-kinase significantly suppressed the lung metastasis of human melanoma cells *in vivo* [73]. NDPK (A or B) is secreted or shed and thus present in the blood stream from various solid and hematological malignancies [49,54,57,74,75]. The disruption of CD39 (ecto-apyrase; EC 3.6.1.5) activity, the dominant vascular ecto-nucleotidase, and its regulation of nucleotide signaling has been observed to inhibit tumor angiogenesis and metastasis [76,77] consistent with, if not directly demonstrative of a role for secreted NM23/NDPK in promoting angiogenesis. We discovered that both MDA-MB-435 and MDA-MB-231 metastatic human breast carcinoma cells secrete NM23/NDPK into their surrounding environment when cultured *in vitro* [57] while the non-metastatic breast cell line MCF-12 does not. Indeed, no less than seven different tumor cell lines established from patients with metastatic breast cancer and two lines developed from primary ductal carcinoma secrete or shed NM23/NDPK into their growth medium in large amounts [78]. Moreover, when immunocompromized SCID mice carry a human breast cancer tumor line (MDA-MB-231) that metastasizes in these animals, NM23/NDPK is found in their blood early on [78,79].

### 2.3. Purinergic Signaling

So if breast cancer cells elaborate NM23/NDPK into their surroundings *in vivo*, what would be the likely consequence [78]? We have proposed that elevation of ATP and ADP in the region of capillary blood vessels by the action of NM23/NDPK would result in activation of purine nucleotide (P2) receptors know to be present on endothelial cells and coupled to vasodilation and angiogenesis [61,63]. The action of nucleotides on endothelium in the region of the tumor cell could promote intravasation. Along with the known ability of ATP receptor stimulation to lead to endothelial cell contraction and thus, vascular permeability [80-82], it is reasonable to consider that maintenance of ATP levels extracellularly by NM23/NDPK is consistent with promotion of transendothelial migration that would subserve intravasation and extravasation.

In the blood stream, ATP and ADP act immediately upon their formation as autocrine and paracrine hormones by stimulating endothelial nucleotide receptors (P2Y_1/2_) to release nitric oxide, prostacyclin and additional ATP [63,83] to dilate arterial resistance vessels. Endothelial ecto-enzyme activities that degrade ATP to ADP [84], together with the tumor cell secreted NM23/NDPK that can regenerate ATP from ADP [63], suggest that nucleotide levels in the relatively acellular boundary layer next to endothelium [85] can be maintained to act at nucleotide receptors. Following its further breakdown, the released purine can finally act again in the venous circulation as adenosine (ADO) where it is the most potent dilator known. ADO dilates the venous circulation to accommodate the increased flow signaled upstream and activates endothelial ADO receptors [86] and inhibits platelet aggregation. These are features of vascular regulation advantageous to the passage of tumor cells in the body and we suggest that the appearance of NM23/NDPK outside the cancer cell whether at the tumor microenvironment for intravasation/extravasation or in the blood stream, subserves dissemination and eventual metastasis.

While the actions of extracellular NM23/NDPK shed by breast cancer cells to take advantage of the vascular actions of ATP is, by itself an important contribution to understanding the biology of breast cancer cells, we have recently expanded on our earlier evidence that activation of the endothelial purinergic receptor signaled in an unexpected manner through the vascular endothelial growth factor (VEGF) receptor [67]. We now know that the effects of endothelial P2Y_1_ receptor stimulation result in the activation of VEGFR-2 in the absence of VEGF [67,78]. This striking result is borne out by the ability of the VEGFR-2 antagonist SU1498 to block the effect of P2Y receptor agonist to cause VEGFR-2 phosphorylation. The enhancement of the effect of NM23/NDPK when a phosphoryl donor and substrate acceptor are present, together with the ability of the NDPK inhibitor ellagic acid [87] to block VEGFR-2 phosphorylation, establishes the effect of NM23/NDPK as acting through P2Y receptor stimulation. The ability of the P2Y_1_ selective agonist 2MeS-ATP to mimic the effect NM23/NDPK, and for both to be prevented by the P2Y_1_R antagonist MRS2179, is convincing proof that NM23/NDPK acts through P2Y_1_R activation in human endothelial cells.

Endothelial cell VEGFR-2 activation by extracellular NM23/NDPK results in Erk_1/2_ phosphorylation which is prevented by ellagic acid, the NM23/NDPK substrate site inhibitor [87], and suramin a non-specific P2YR antagonist. Together these results indicate that activation of P2Y receptors by extracellular NM23/NDPK is crucial in the transactivation of VEGFR-2 and subsequent down-stream regulation of the Raf-MEK-MAPK pathway that regulates growth and migration of endothelial cells [88].

### 2.4. Nucleotide-Mediated VEGFR Activation

The ability of the inhibitor (PP2) of the proto-oncogene non-receptor tyrosine kinase, Src to block both P2Y_1_R and VEGF stimulation of VEGFR-2 phosphorylation, while having little effect on VEGF-stimulated Erk_1/2_ phosphorylation is consistent with the notion that P2Y_1_R signals to activate VEGFR-2 via *Src* activation. The ability of VEGF to activate VEGFR-2 Tyr-1175 phosphorylation is, by contrast, a direct effect of the growth factor to bind to and activate its receptor. The contrast in the transactivation of VEGFR-2 by P2Y_1_R activation via the action of NM23/NDPK *versus* VEGF activation can be seen in the ability of PP2 to block P2Y_1_R but not VEGF activation of VEGFR-2 [78]. The Src inhibitor PP2 fails to prevent VEGF activation of Erk_1/2_ confirming that Src does not phosphorylate Erk in human endothelial cells. The notion established by these data is that VEGF antagonists alone may not perform well clinically as angiogenesis supressors since nucleotide activation of VEGFR would be unaffected. Tyrosine kinase inhibitors have demonstrated clinical utility in inhibiting VEGF receptor, epidermal growth factor receptor, and platelet-derived growth factor receptor [89]. However, all of these anti-angiogenic therapies tend to exhibit low response rates when used as a monotherapy [90], are astonishingly expensive [91,92] and resistance to these agents tends to build quickly [93].

We have previously shown that the activation of the P2Y_1_ receptor is crucial in the activation of tubule formation in endothelial cells [61] *in vitro*. Our more recent examination of the mechanisms of the P2Y_1_ receptor pathway with regard to transactivation of VEGFR-2 Tyr1175, extracellular signal-related kinase (Erk_1/2_) activation, and functional determination of cell growth and migration, reinforce the biology of the extracellular actions of NM23/NDPK in support of tumor angiogenesis [78]. The ability of VEGF to stimulate endothelial cell growth is mimicked by NM23/NDPK in a concentration dependent manner. Endothelial cell proliferation is prevented by ellagic acid consistent with the known ability of ellagic acid to block NM23/NDPK activity. These data further enforce the importance of extracellular NM23/NDPK in breast tumor biology.

Recent studies from Cynthia Bamdad and colleagues have focused attention on NM23/NDPK and a heretofore unrecognized binding partner, Muc-1. The mucin glycoprotein normally expressed at the apical border of epithelia (MUC1) is overexpressed by breast cancer cells [94,95] that release/shed a high molecular weight fragment (Muc-1) in the conditioned medium from breast cancer cells in culture [96]. Muc-1 apparently binds NM23/NDPK [97] and is proposed to activate the MAP-kinase signaling pathway and stimulate growth. The finding that purified bovine NDPK binds to Muc-1 was seen as evidence for its growth stimulatory properties in human embryonic stem cells [98]. These data fit well with our own [67,78] and further emphasize an extracellular role for NM23/NDPK. In the studies described here and elsewhere [2], NDPK is acting via P2Y receptor activation in an ATP-dependent manner. A role for the released Muc-1 protein in the breast cancer angiogenic process is interesting since it would mediate actions of NDPK acting directly by increasing and/or directing its association and/or decreasing its diffusion from sites of release and as such fits with our overall hypothesis regarding the importance of NM23/NDPK.

## 3. Conclusions

The evidence that we need to revisit our understanding of breast cancer without the bias of our assumptions about uncontrolled growth of tumors as a hallmark of the disease is sorely needed. The fact of dormancy demands that we examine tumor biology in the context not just of the properties of tumor cells themselves, but of the environment in which they move in the body and hijack normal mechanisms. It is time too that we lose the dogmatic assumptions of the associations of genes with metastatic potential as causative or singularly instructive to further research. A case in point is NM23/NDPK. Earlier work that has branded this protein in one or more of its forms, as a metastasis suppressor from gene expression studies, completely ignores its extracellular actions and biases the field to other ideas. Indeed, the transactivation of the vascular endothelial growth factor receptor via NM23/NDPK activation of human endothelial purinergic receptors offers a framework for the dramatic ability of the nucleotide pathway to promote cancer cell dissemination and angiogenesis. Studies to link Muc-1, NDPK, inflammatory cells, the endothelial cell and its P2Y receptors may further our understanding of tumor cell mediated metastasis.

In the end, what matters to women is what we can learn to treat their disease more effectively. What matters in the laboratory is that we can advance our ideas to some reasonable measure of effectiveness in *in vivo* models since none of this will be useful if it cannot lead to new ways of treating breast cancer. Translation is essential. Breast cancer research cannot afford the luxury of eccentricity. This translation is ongoing now for the actions of NM23/NDPK described here. We have collected evidence at the time of writing that metastasis of MDA-MB-231 cells in immunocompromized SCID mice is significantly lessened (completely prevented in our first experimental series) by treating animals with NM23/NDPK inhibitor and an antagonist of the endothelial P2Y receptor. Breast cancer has eluded our efforts to prevent it for centuries. Perhaps it is time to look beyond what has been assumed, or learned from other diseases and be led by the data, no matter how inconvenient that may be for the field.
